# α-Synuclein and Noradrenergic Modulation of Immune Cells in Parkinson’s Disease Pathogenesis

**DOI:** 10.3389/fnins.2018.00626

**Published:** 2018-09-11

**Authors:** Laura M. Butkovich, Madelyn C. Houser, Malú G. Tansey

**Affiliations:** Tansey Laboratory, Department of Physiology, School of Medicine, Emory University, Atlanta, GA, United States

**Keywords:** α-synuclein, locus coeruleus, Parkinson’s disease, neuroinflammation, norepinephrine, immune cell

## Abstract

α-synuclein (α-syn) pathology and loss of noradrenergic neurons in the locus coeruleus (LC) are among the most ubiquitous features of Parkinson’s disease (PD). While noradrenergic dysfunction is associated with non-motor symptoms of PD, preclinical research suggests that the loss of LC norepinephrine (NE), and subsequently its immune modulatory and neuroprotective actions, may exacerbate or even accelerate disease progression. In this review, we discuss the mechanisms by which α-syn pathology and loss of central NE may directly impact brain health by interrupting neurotrophic factor signaling, exacerbating neuroinflammation, and altering regulation of innate and adaptive immune cells.

## Introduction

Locus coeruleus (LC) degeneration and α-synuclein (α-syn) aggregation are among the most ubiquitous features of Parkinson’s disease (PD) (Chui et al., 1986; German et al., 1992; Zarow et al., 2003). Brain regions affected in PD, including the LC, contain large protein-rich intracellular inclusions known as Lewy bodies (LB) or Lewy neurites (LN) accompanied by chronic inflammation and neuron loss (den Hartog and Bethlem, 1960; Spillantini et al., 1997; Tansey and Goldberg, 2010). While LBs and LNs contain numerous proteins, α-syn is the predominant component (Spillantini et al., 1997), and α-syn is the major pathological protein underlying PD pathogenesis. α-syn is a 140-amino acid protein encoded by the *SNCA* gene, which is expressed in many tissue types and which accounts for approximately 1% of cytosolic proteins in neurons (Shibayama-Imazu et al., 1993; Iwai et al., 1995; Stefanis, 2012). It is highly expressed in the presynaptic terminals where it acts as a molecular chaperone in SNARE formation and vesicular trafficking (Burre et al., 2010). Genetic evidence comes from individuals carrying *SNCA* mutations, which confer increased risk of PD, or autosomal dominant forms of PD (Klein and Schlossmacher, 2006). Finally, animal models overexpressing α-syn develop age-dependent α-syn aggregates and PD-like behavioral abnormalities (Masliah et al., 2000; Giasson et al., 2002). The initiating event in α-syn aggregation is unknown, but Lewy pathology (LP) and cell loss are common within discrete neuronal populations in PD.

Extensive dysfunction of catecholaminergic neurons is a well-established feature of PD, and a major hallmark is LP and loss of dopaminergic (DA) neurons in the substantia nigra pars compacta (SNpc) which induces motor impairments including tremor, muscle rigidity, bradykinesia, and postural instability (Hirsch et al., 1988; Fearnley and Lees, 1991; Parkinson, 2002). A diagnosis of PD is currently dependent on the presence of motor symptoms and striatal dopamine deficiency; however, PD is a multifactorial disease with non-motor symptoms that are associated with alterations in cholinergic, serotonergic, and noradrenergic systems occurring years or even decades prior to the onset of motor dysfunction (Gonera et al., 1997; Abbott et al., 2005; Ross et al., 2008). LP and degeneration of several pontine and medullary nuclei (including the dorsal raphe, dorsal motor nucleus of the vagus, pedunculopontine nucleus, and LC) are ubiquitous features of PD (Halliday et al., 1990). The LC is the major source of norepinephrine (NE) to the CNS, and it is among the first brain regions to be affected in PD (Iversen et al., 1983; Mann and Yates, 1983; Braak et al., 2001). NE is the ligand for the adrenergic receptors (ARs) comprised of seven G-protein coupled receptors that are G_q_-, G_i/o_-, or G_s_-coupled, allowing NE to have diverse functional effects dependent on receptor expression and cell type (Strosberg, 1993). LC neurons are constitutively active and innervate virtually every brain region via extensive and complex axonal arborization that facilitates the release of both synaptic NE and extra-synaptic NE at axonal varicosities (Freedman et al., 1975; Grzanna and Molliver, 1980; Jones and Yang, 1985; Agnati et al., 1995) where LC-NE can be neuroprotective by both direct and indirect mechanisms. Here, we will review evidence that LC dysfunction may exacerbate PD pathophysiology and may represent a tipping point in disease progression.

## LC-NE Dysregulation Could Promote the Progression of PD Pathology

It is unclear why certain neuronal populations like the LC are vulnerable to α-syn pathology, but sensitivity to oxidative stress, pacemaker activity, and extensive contact with blood vessels that may expose LC neurons to circulating toxins have been implicated (Jenner, 2003; Cho, 2014; Pamphlett, 2014). The degree of noradrenergic innervation to a brain region is negatively correlated with DA loss (Tong et al., 2006), indicating that the loss of central NE and its neuroprotective actions may directly influence the rate of PD progression. In PD, loss of LC neurons begins prior to nigral pathology and appears to be of greater magnitude (German et al., 1992; Zarow et al., 2003; Szot et al., 2006; Brunnstrom et al., 2011). Per the Braak staging hypothesis of PD pathology, LP first appears in brainstem nuclei (stage 1), and, as PD progresses, continues along a caudo-rostral axis with LC pathology appearing at stage 2 and SNpc pathology at stage 3, before ultimately extending into cortical regions (Braak et al., 2003). PD brain tissue has marked LC denervation in many brain regions and loss of LC cell bodies that extends throughout its rostral-caudal axis (Javoy-Agid et al., 1984; German et al., 1992; Pavese et al., 2011). Imaging and postmortem histological studies of PD patients reveal a progressive loss of central NE throughout the brain (Pifl et al., 2012) along with accumulation of α-syn and loss of LC neurons (Halliday et al., 1990; Chen et al., 2014; Keren et al., 2015; Isaias et al., 2016). LC neuron vulnerability to α-syn pathology can be replicated experimentally. A recent model targeted viral vector-mediated overexpression of a familial PD mutant α-syn variant to the murine LC region (Henrich et al., 2018). While transgene expression was not restricted to neuronal cells, the resulting progressive α-syn aggregation, gliosis, and LC degeneration are reminiscent of LC pathology found in PD. Enzymes responsible for NE synthesis and NE metabolite levels are reduced in the CSF of PD patients, also supporting these central changes in NE metabolism (Hurst et al., 1985; Goldstein et al., 2012). Evidence of early LC dysfunction can be found in patients who do not meet the diagnostic criteria for PD. In such individuals, decreased neuron density in the LC, but not VTA or dorsal raphe, corresponds to the severity of global parkinsonism (Buchman et al., 2012), suggesting that this state may represent prodromal/preclinical PD. Patients who had LP at autopsy but lacked any of the clinical signs of PD also had reduced LC neuron density as compared to DA neurons in the SNpc, further highlighting the possible early role of LC neuron loss in PD (Dickson et al., 2008).

There is also evidence that α-syn may directly affect NE homeostasis by two separate mechanisms. First, norepinephrine transporter (NET)-expressing cells transfected for α-syn expression reveal that high levels of α-syn negatively regulate NET expression on the cell surface, while relatively lower levels increase NET expression (Wersinger et al., 2006). Second, when α-syn is overexpressed in an NE-producing cell line or transgenic rodent model, it can translocate to the nucleus and directly interfere with transcription of dopamine ß-hydroxylase (DBH), the enzyme involved in the final step of NE synthesis, reducing NE production (Kim et al., 2011, 2014). It is possible that interfering in NE neurotransmission could, in turn, impact α-syn expression as ß-adrenergic receptor (ß-AR) agonists reduce *SNCA* mRNA and α-syn protein expression in induced pluripotent stem cells derived from individuals carrying the *SNCA* triplication mutation (Mittal et al., 2017). Together, these data indicate that α-syn can influence NE metabolism, and that this, in turn, could impact α-syn expression, although additional work is required to determine if this is clinically relevant.

## PD Non-Motor Symptoms

The LC is the major source of NE to the CNS (Mouton et al., 1994), and dysregulated noradrenergic innervation is associated with many of the non-motor symptoms of PD including anxiety (Casacchia et al., 1975; Stein et al., 1990; Nuti et al., 2004), depression (Shulman et al., 2002; Ravina et al., 2007), rapid eye movement (REM) sleep behavioral disorder (RBD) (Sixel-Doring et al., 2011; Kalaitzakis et al., 2013), and dementia (Chui et al., 1986).

Up to 60% of PD patients report experiencing some form of anxiety (Chaudhuri and Schapira, 2009; Lin et al., 2015; Houser and Tansey, 2017). Dopamine, serotonin, and NE have been implicated in PD anxiety, suggesting that its neurobiological origins are complex (Eskow Jaunarajs et al., 2011; Thobois et al., 2017; Joling et al., 2018). LC neurons are highly active during stress exposure (Bingham et al., 2011; Curtis et al., 2012) and innervate all corticolimbic regions involved in the anxiety response (Aston-Jones et al., 1991, 1999). In PD patients, anxiety severity is inversely correlated with dopamine/NE transporter binding in the LC (Remy et al., 2005), and experimentally, selectively inhibiting LC neurons during stress exposure blocks the subsequent anxiety-like behavior (McCall et al., 2015).

Around 35% of PD patients suffer from depression (Reijnders et al., 2008; Houser and Tansey, 2017). Dysfunction of LC-NE is known to be associated with depression (Moriguchi et al., 2017) and is a common pharmacological target in the treatment of depression (Ressler and Nemeroff, 2001; Remy et al., 2005). Indeed, early investigation of NET expression in the LC reported decreased NET in major depressive disorder (Klimek et al., 1997), although results from subsequent studies have been inconsistent (Moriguchi et al., 2017). While it is unclear if NET is downregulated due to lack of available NE or in order to increase synaptic NE levels, it is clear that NE dysfunction can contribute to depressive symptoms.

LC neuron activity fluctuates diurnally with increased activity immediately prior to waking and during waking hours (Hobson et al., 1975). Sleep disturbances are one of the most common complaints from PD patients (Smith et al., 1997) and can include insomnia (Gjerstad et al., 2007), excessive daytime sleepiness (Rye et al., 2000), and RBD (Comella et al., 1998; Gagnon et al., 2002). A recent study reported that disturbed sleep is positively correlated with anxiety and depression in PD (Rana et al., 2018). In fact, RBD is the most predictive non-motor symptom of synucleinopathies with up to 92% of idiopathic RBD patients receiving a synucleinopathy diagnosis within 14 years (Iranzo et al., 2006; Postuma et al., 2009; Schenck et al., 2013). There is evidence that LC neurons in individuals that have PD with disturbed sleep contain more LP than in those without (Kalaitzakis et al., 2013), and mice lacking DBH (and subsequently, NE) have significantly disturbed sleep behavior (Hunsley and Palmiter, 2003). Together, these data suggest that loss of central NE may directly contribute to the development of sleep disturbances in PD.

An estimated 83% of PD patients will experience some sort of cognitive dysfunction, including dementia (Hely et al., 2008). Dementia is characterized by cognitive impairment, including memory loss, attentional deficits, and loss of executive function (Elizan et al., 1986; Aarsland et al., 2003). While dementia is generally associated with cholinergic deficits and late-stage PD, early executive disturbances may arise from deregulation of LC-NE. PD patients with dementia have more extensive loss of LC-NE in cortical regions than those without (Chan-Palay and Asan, 1989). In fact, degeneration of LC neurons and loss of cortical NE is a central component of dementia of Alzheimer’s type (Mann and Yates, 1983; Zarow et al., 2003). In animal models, hippocampal LC-NE is essential for proper memory acquisition and retrieval (Devauges and Sara, 1991; Mello-Carpes et al., 2016), and loss of LC neurons can impact memory and enhance cognitive deficits (Ohno et al., 1997; Chalermpalanupap et al., 2018).

## Beyond the Non-Motor Symptoms

The temporal relationship between LC and SNpc pathology suggests that loss of LC-NE may leave SNpc neurons more vulnerable to α-syn toxicity and potentiate the rate of PD progression. Experimentally, loss of LC-NE exacerbates 6-OHDA- and MPTP-mediated nigral degeneration in rodent and primate models (Mavridis et al., 1991; Srinivasan and Schmidt, 2003; Rommelfanger et al., 2007; Yao et al., 2015), while increasing synaptic NE by genetic deletion or pharmacological blockade of the NE transporter (NET) confers resistance (Kilbourn et al., 1998; Rommelfanger et al., 2004). Indeed, individuals with a functional polymorphism in the promoter regions of the *DBH* gene have reduced risk of developing PD (Healy et al., 2004). In sum, these data demonstrate that loss of NE may exacerbate nigral pathology.

## Neuroprotective Effects

NE can directly act as a neurotrophic factor but can also indirectly stimulate neurotrophic factor expression. Primary mesencephalic cultures treated chronically with NE have a significantly reduced rate of cell death, increased neuritic processes, and reduced production of reactive oxygen species when compared to untreated cultures, and this phenotype resembles cultures treated with traditional antioxidants (Troadec et al., 2001, 2002). Increasing synaptic NE was shown to be protective against neuron loss and inflammation in a model of hypoxic-ischemia (Toshimitsu et al., 2018). While NE ligation of ARs directly facilitates neuroprotection by several mechanisms, the neuroprotective effects are not always blocked by AR antagonists, suggesting NE-mediated protection may also occur indirectly. One candidate mechanism of interest is the neuropeptide brain-derived neurotrophic factor (BDNF), which is synthesized and released by astrocytes and neurons, including those in the LC (Castren et al., 1995). BDNF signaling is primarily mediated by binding to the high affinity tropomyosin-related kinase B receptor (TrkB), which can protect SNpc neurons in experimental models, and BDNF mRNA is reduced in the SNpc in PD (Hyman et al., 1991; Spina et al., 1992; Howells et al., 2000). NE can also enhance BDNF transcription and BDNF/TrkB kinetics (Chen et al., 2007). Activation of the β1-adrenergic receptor stimulates BDNF transcription in astrocytes (Koppel et al., 2018). When BDNF binds to TrkB, signal transduction is mediated by TrkB dimerizing and autophosphorylating (Haniu et al., 1997). NE can induce autophosphorylation of TrkB and is protective against cell death in primary culture (Liu et al., 2015). In addition to loss of NE, α-syn may also directly disrupt the neuroprotective effects of BDNF. A recent study demonstrated that α-syn has the potential to bind the kinase domain on TrkB receptors, preventing the neurotrophic signaling of BDNF/TrkB, and that this exacerbates degeneration of DA neurons (Kang et al., 2017).

## Central Inflammation

Neuroinflammation is a vital mechanism in restoring brain integrity following neuronal insult but is also a core component of PD pathology. In a healthy brain, the inflammatory response resolves relatively quickly, with normal brain function restored (Roth et al., 2014; Laumet et al., 2018). In neurodegenerative diseases, such as PD, sustained neuroinflammation can become cytotoxic, aggravating neuronal degeneration. It is unclear what triggers the initial inflammation in PD, but extracellular monomeric or aggregated α-syn can be phagocytosed by microglia and induce their activation (Zhang et al., 2005; Hoenen et al., 2016), and neuronal overexpression of α-syn aggravates and prolongs neuroinflammation (Miller et al., 2007; Gao et al., 2011; Sanchez-Guajardo et al., 2013). In PD patients, immune mediators such as IL-1ß, TGFß, IFNγ, and IL-6 are increased in the cerebral spinal fluid (CSF) and nigrostriatal regions (Mogi et al., 1994; Blum-Degen et al., 1995; Mount et al., 2007), and SNpc DA neurons appear particularly sensitive to pro-inflammatory cytokines (McGuire et al., 2001; Mount et al., 2007; Tansey and Goldberg, 2010). In fact, neuroinflammation is detectable prior to signs of neuronal degeneration, suggesting a potential early role for inflammation in PD pathogenesis (Theodore et al., 2008; Watson et al., 2012).

Research indicates that dysregulation of noradrenergic signaling may also play a role in driving inflammation. Like overexpression of neuronal α-syn, lesioning LC neurons using a noradrenergic-specific toxin also induces inflammation (Theodore et al., 2008; Watson et al., 2012; Yao et al., 2015; Song et al., 2018). NE can have activating or inhibitory effects on immune cells depending on adrenergic receptor expression, which varies depending on the cellular environment (Khan et al., 1985; Tanaka et al., 2002). Therefore, LC degeneration and subsequent deficient brain NE may contribute to PD pathology by loss of normal immune cell modulation. Microglia, the brain-resident macrophages, are the sentinels of brain parenchyma, monitoring tissue integrity and responding to infection or injury (Nimmerjahn et al., 2005). When ramified (resting) microglia are activated, they adopt an amoeboid morphology, proliferate, and become phagocytic, releasing pro-inflammatory cytokines which can recruit central and peripheral immune cells to the site of insult (Hayes et al., 1987). There is extensive evidence of sustained microglial over-activation in degenerating brain regions in PD (Kim and Joh, 2006; Tansey and Goldberg, 2010), and inhibiting microglia activation with minocycline prevents DA neuronal loss in mice treated with a DA neuron-specific toxin (Wu et al., 2002).

Microglia express many neurotransmitter receptors, including ARs (Pocock and Kettenmann, 2007). While more studies are required to understand how AR activation affects microglial phenotypes, depletion of NE, as is found in PD, exacerbates microglial inflammatory responses (Heneka et al., 2002; Bharani et al., 2017). AR-mediated modulation of microglia is well documented, although reports of the functional outcome are inconsistent. In murine brain slices, resting microglia appear to preferentially express the excitatory ß2-AR, but shift toward the inhibitory α2-AR receptor expression following activation with the canonical microglial activator lipopolysaccharide (LPS) (Gyoneva and Traynelis, 2013). However, microglial treatment with an ß2-AR agonist is reported to have anti- or pro-inflammatory effects. For example, cultured primary microglia treated with a ß2-AR agonist suppressed microglial proliferation (Fujita et al., 1998), while a subsequent study reported that priming microglia with a ß2-AR agonist prior to LPS treatment significantly increased pro-inflammatory IL-1ß and IL-6 expression (Johnson et al., 2013). The functional outcome of microglial AR activation appears dependent on the physiological context, and further examination is needed to determine how this may influence PD pathology.

## Peripheral Inflammation

There is abundant evidence that the inflammatory manifestations of PD are not confined to the CNS. Indicators of inflammation have been found in the colon tissue, stool, and blood as well as in the CSF. Colonic expression of the genes encoding pro-inflammatory cytokines TNF, IFNy, IL-6, and IL-1β is increased in PD, accompanied by evidence of gliosis (Devos et al., 2013). Recently, we reported that IL-1α, IL-1β, CXCL8, and CRP are significantly elevated in stool from PD patients compared to controls (Houser et al., 2018), and that serum levels of TNF, IFNy, and neutrophil gelatinase-associated lipocalin levels are significantly and consistently different in PD over a 24-h period (Eidson et al., 2017). Local α-syn expression has been found to increase under inflammatory conditions in the periphery (Stolzenberg et al., 2017), and α-syn pathology has been observed in the enteric nervous system of PD patients, even from the earliest stages of disease (Stokholm et al., 2016; Barrenschee et al., 2017; Punsoni et al., 2017). These findings demonstrate that similar pathological processes are active in the CNS and the periphery in PD, and there is almost certainly significant crosstalk between them.

Degradation of the blood-brain-barrier (BBB) has been well documented in PD (Kortekaas et al., 2005; Pisani et al., 2012; Gray and Woulfe, 2015), and it has been proposed that this impaired barrier function exposes the CNS to circulating factors that could promote α-syn aggregation (Gray and Woulfe, 2015), immune cell infiltration, neuroinflammation, and, ultimately, neurodegeneration (Rite et al., 2007). Whether through direct effects of reduced signaling through endothelial β-ARs or through increases in vascular permeability-promoting inflammation, LC neurodegeneration compromises the integrity of tight junctions (Kalinin et al., 2006) and increases permeability of the BBB (Nag and Harik, 1987). BBB leakiness enables greater interaction between central and peripheral immune activities, allowing exchange of cytokines, chemokines, and other circulating molecules and potentially even facilitating infiltration of peripheral immune cells into the CNS where loss of central NE modulation could result in aberrant immune cell activity.

As with brain-resident microglia, immune cells originating in the periphery can also be modulated by NE. Peripheral immune cells infiltrate the brain parenchyma in PD (Kannarkat et al., 2013), and these will likely be directly impacted by reduced levels of central NE. Peripheral NE levels may also play important immunomodulatory roles in PD. The NE deficiency found in the CNS in PD is not consistently recapitulated in the periphery, with several studies reporting no difference in NE levels in plasma from PD patients compared to healthy controls (Eldrup et al., 1995; Goldstein et al., 2003). It is likely, however, that at least a subset of PD patients is affected by peripheral NE dysregulation as evinced by the prevalence of neurogenic orthostatic hypotension (NOH) associated with this disease. NOH is a condition in which insufficient noradrenergic activity results in failure to appropriately increase blood pressure (BP) in response to a postural change such as sitting up or standing. This results in insufficient cerebral blood supply and can produce lightheadedness and dizziness, which increase fall risk (Merola et al., 2016). NOH occurs frequently in conditions involving synucleinopathy, and roughly 30% of PD patients are affected. NOH in PD is attributed to noradrenergic postganglionic sympathetic denervation associated with LP and a subsequent failure to induce sufficient NE production when transitioning to an upright position (reviewed by Loavenbruck and Sandroni, 2015). PD patients with orthostatic hypotension exhibit lower levels of NE in plasma compared to PD patients without NOH that reach levels significantly lower than non-PD controls (Senard et al., 1990; Niimi et al., 1999; Goldstein et al., 2005). This creates the potential for PD-associated NE deficiency to modulate peripheral immune responses as well as central.

Nearly every lymphoid tissue in the body has postganglionic sympathetic innervation, and peripheral innate and adaptive immune cells express ARs, rendering them responsive to NE. Excitatory β2-ARs are the most highly expressed ARs on peripheral immune cells, and their activity likely dominates the immune response to NE. β-AR signaling has potent anti-inflammatory effects on innate immune cells (reviewed by Qiao et al., 2018). In macrophages, which bear close functional resemblance to microglia, it suppresses pro-inflammatory activity and promotes tolerogenic and homeostatic phenotypes (Grailer et al., 2014; Noh et al., 2017). It also limits the number and the effector functions of natural killer (NK) cells (Whalen and Bankhurst, 1990; Takamoto et al., 1991). Adrenergic signaling has been shown to impair the functions of neutrophils and eosinophils as well (Gosain et al., 2009; Brunskole Hummel et al., 2013; Noguchi et al., 2015). Dendritic cells connect the innate and adaptive immune responses by sampling antigens in the local environment and then presenting them with appropriate polarization signals to T cells. β2-AR activation profoundly suppresses dendritic cell functionality, inhibiting their maturation, migration, antigen presentation including cross presentation, and proinflammatory cytokine production while inducing expression of anti-inflammatory factors (Seiffert et al., 2002; Herve et al., 2013; Chen et al., 2016; Qiao et al., 2018). It is important to note that while these anti-inflammatory effects on innate immune cells are well-documented, study designs differ widely, and the effects they observe on these cells vary depending on physiological context, time, AR agonist, and dose. Further research will be necessary to better characterize the relationship between NE and innate immune responses.

CD4+ T helper (Th) cells are indirectly affected by AR agonists due to their suppressive effects on dendritic cells which result in diminished differentiation of effector T cells, particularly Th1s (Wu et al., 2016). Th1 cells also express β2-ARs (McAlees et al., 2011), and their proliferation and activity are inhibited upon ligation of this receptor (Ramer-Quinn et al., 1997; Riether et al., 2011). Since Th2 cells do not express ARs (McAlees et al., 2011), their functionality is not directly modulated by exposure to NE, but NE-mediated suppression of Th1 cells would relieve their negative regulatory pressure on Th2 cells, indirectly promoting Th2-mediated immune activity, which is canonically involved in anti-helminth and allergic immune responses but not classic inflammation (Huang et al., 2015). β2-AR signaling also impairs the activity of CD8+ memory and effector T cells (Chen et al., 2016; Estrada et al., 2016; Bucsek et al., 2017).

The consequences of AR ligation on other T cell subsets are less straightforward. The intricacies of the potential effects of NE on CD4+ Th17 cells are just beginning to be elucidated. These cells are important actors in normal mucosal immunity, but they are also implicated in autoimmune pathology. Several studies have reported that treatment of CD4+ cells with NE promotes differentiation of Th17 cells and increases their activity (IL-17 production) while simultaneously inhibiting Th1 differentiation and activity (IFNγ production) (Carvajal Gonczi et al., 2017; Xu et al., 2018). On the other hand, studies of Th17 cells from both mice and humans with Th17-mediated autoimmune diseases found that treating CD4+ T cells with NE inhibited the differentiation and activity of Th17 cells (IFNγ production was also still reduced) (Boyko et al., 2016; Liu et al., 2018). This indicates that the immunoregulatory effects of NE on Th17 cells are dependent on the physiological context. It is also possible that autoimmune conditions in which pathology is mediated in part by IL-17-producing cells might constitute a unique context in which this alternative regulatory action of NE is observed. For instance, in such conditions, a highly inflammatory cell type that exhibits characteristics of both Th1 and Th17 cells is typically present (Murphy et al., 2010), and it may be that the actions of NE on this particular cell type rather than on canonical Th17s dominate its observed effects in these autoimmune diseases.

Findings on NE modulation of CD4+ T regulatory (Treg) cells, an anti-inflammatory subset which counteracts effector functions of other types of T cells, are even more ambiguous. One study reports that treatment of Tregs with NE prior to transfer in an autoimmune arthritis mouse model rendered them pathological and worsened the disease (Harle et al., 2008). In the same vein, another study found that NE exposure decreased the regulatory activity of Tregs and even induced their apoptosis (Wirth et al., 2014). On the other hand, a study in humans reported that Treg frequencies were elevated under conditions which increased circulating NE levels and that treatment of Tregs with epinephrine, which is chemically similar to NE and binds the same receptors, stimulated Treg proliferation. This effect was blocked by treatment with a β-AR antagonist (Inoue et al., 2017). A final study reported no detectable effects of treatment with NE or epinephrine on human Tregs, though they did determine that they could express three different types of ARs (Cosentino et al., 2007). Obviously, more research is needed to determine the effect of NE on Tregs.

B cells also express β2-ARs, and there is evidence that NE can negatively regulate the magnitude of antibody responses. The effects are highly varied, however, as they are influenced by the effects of NE on T cells, by the stimuli used to activate B cells, and by the immunological and physiological context of the experiment (extensively reviewed by Kin and Sanders, 2006). A couple of more recent studies suggest that, under conditions of autoimmune disease in which B cells contribute to inflammatory activity and pathology, NE exerts a suppressive effect on these cells which is mediated by decreased IL-7 receptor signaling and enhanced production of anti-inflammatory IL-10 (Pongratz et al., 2012, 2014).

The effects described here do not represent the full extent of peripheral NE-mediated neuroimmune interactions. Most studies to date have focused on the results of β2-AR signaling, but immune cells express other ARs as well which can mediate different effects (Lorton and Bellinger, 2015), and, as in the brain, the relative levels of these receptors change in different immune environments. Activation of the same AR can even produce distinct responses depending on the concentration of the ligand and its temporal relationship to immunogenic stimuli (reviewed by Lorton and Bellinger, 2015). This provides important plasticity for neuroimmune regulatory mechanisms. Nonetheless, many functional studies support the existing literature that indicates a primarily anti-inflammatory impact of peripheral NE. Vagus nerve stimulation is known to have clear immunosuppressive effects (Inoue et al., 2017) and to reduce synuclein expression in the brain (Farrand et al., 2017), and these effects are mediated in large part by NE signaling through β-ARs (Vida et al., 2011). A recent review (Bucsek et al., 2018) summarized numerous studies showing that chemical ablation of sympathetic neurons or β-AR blockade enhanced immune responses to different bacterial, viral, and parasitic infections while AR agonist treatment impaired anti-viral and anti-parasite responses. Several of the studies found that these effects were specific to modulation of peripheral adrenergic activity, but it was also demonstrated that this could induce corresponding immune responses in the CNS. Similarly, another study found that ablation of peripheral and LC noradrenergic neurons prompted an exaggerated acute inflammatory response to peripheral LPS that was observed both in the brain and in the circulation (Bharani et al., 2017).

Taken together, the data on peripheral immune cells and their function when challenged indicate that NE is immunosuppressive, and as such, postganglionic sympathetic denervation and NE deficiency in PD could stimulate pro-inflammatory immune activity. This has implications for PD pathogenesis and the progression of disease pathology. Peripheral and systemic inflammation have been well documented in PD, and it has been proposed that inflammatory mechanisms may contribute to non-motor symptoms and also be responsible for the development and spread of synucleinopathy and the induction of neuroinflammation and neurodegeneration in this disorder (Qin et al., 2016; Houser and Tansey, 2017). PD-associated gastrointestinal abnormalities and dysfunction are consistent with inflammatory conditions in the gut (Houser and Tansey, 2017), and levels of proinflammatory cytokines in the blood correlate positively with the severity of anxiety and depression in PD patients (Wang et al., 2016). α-syn levels increase in the context of immune activation, and some data suggest that peripheral inflammation can induce elevated α-syn expression in the brain (Kelly et al., 2014) and that peripheral α-syn can migrate to the brain through the vagus nerve (Holmqvist et al., 2014). α-syn has also been shown to exert chemoattractant properties on peripheral myeloid cells, including recruiting them into the brain in a rodent PD model (Stolzenberg et al., 2017; Harms et al., 2018). Infiltration of peripheral CD4+ and CD8+ T cells into the brain has also been observed in PD (Brochard et al., 2009), and it has been shown that these T cells (primarily the CD4+ subset) in peripheral blood from PD patients recognize and respond to peptides derived from α-syn (Sulzer et al., 2017). In animal models of parkinsonian neuropathology, invading monocytes and CD4+ T cells have been identified as key mediators of neurodegeneration (Brochard et al., 2009; Harms et al., 2018).

NE deficiency, centrally and/or in the periphery, could potentiate all of these immune-mediated effects in PD. It would impair anti-inflammatory regulatory functions, shifting immune cells toward more pro-inflammatory phenotypes. Innate immune cells affected in this way would be less able to clear α-syn aggregates and neuronal debris effectively and in a toleragenic manner and more likely to recruit additional effector cells, stimulate their pro-inflammatory activities, and perhaps even present α-syn and other neuronal antigens in a context which could induce autoimmune responses (Sulzer et al., 2017). Furthermore, the activity of at least some T cell subsets which may be pathologically involved in PD could be potentiated by a loss of inhibitory NE signaling. Especially in the context of a compromised BBB, these pro-inflammatory immune cells and their products would have greater access to the CNS and could infiltrate and mediate damaging effects on neurons there.

## Discussion

**FIGURE 1:**
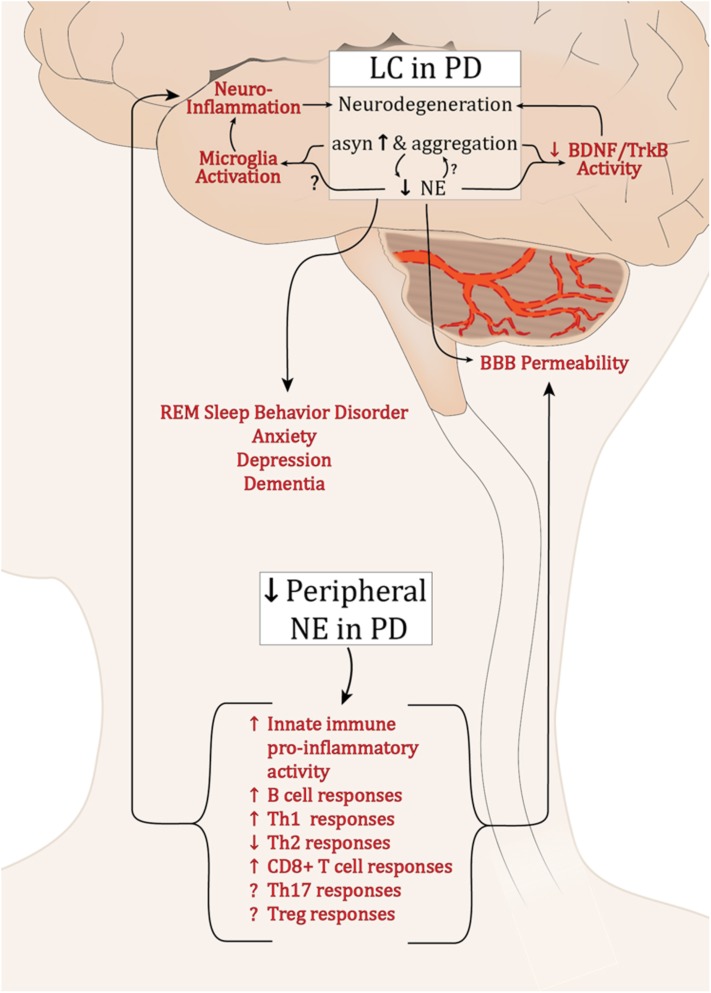
Potential effects of dysregulated NE in PD. Abbreviations: α-syn, α-synuclein; BBB, blood brain barrier; B cell, B lymphocytes; BDNF, brain derived neurotrophic factor; LC, locus coeruleus; PD, Parkinson’s disease; NE, norepinephrine; Th1, T helper 1 lymphocytes; Th17, IL-17 producing T helper lymphocytes; Th2, T helper 2 lymphocytes; CD8+, cytotoxic T lymphocytes; Treg, T regulatory lymphocytes; Trkb, tropomyosin-related kinase B receptor.

Extensive dysfunction of catecholaminergic neurons is a well-established feature of PD, and while a major hallmark is LP and loss of DA neurons in the SNpc, PD is a multifactorial disease with alterations in cholinergic, serotinergic, and noradrenergic systems occurring years earlier and generally associated with PD’s non-motor symptoms (Halliday et al., 1990; Braak et al., 2003). α-syn pathology and a progressive decline in LC-NE have been well characterized; still it is unclear why these neurons are among the most vulnerable in PD. Still less is known about how the deficits in LC-NE and the loss of its neuroprotective and neuroimmune modulatory effects could influence the development of synucleinopathy and exacerbate PD pathology (summarized in **Figure 1**). Preclinical research has provided compelling evidence supporting the neuroprotective functions of NE. Experimentally, depletion of NE renders SNpc neurons vulnerable in toxin models of PD (Mavridis et al., 1991; Srinivasan and Schmidt, 2003; Rommelfanger et al., 2007), while NE enhancement is protective (Kilbourn et al., 1998; Rommelfanger et al., 2004). Additionally, there is a reciprocal modulatory relationship between α-syn and NE whereby α-syn can modulate NE neurotransmission, both at the level of synthesis (Kim et al., 2014), and by modulating NET expression at the cell surface (Wersinger et al., 2006), and NE can attenuate *SNCA* transcription and α-syn protein expression (Mittal et al., 2017). As PD pathophysiology progresses, LP develops in the SNpc and other brain regions, and LC-NE denervation may exacerbate the rate and/or degree of degeneration during this premotor phase of PD. Experimentally, NE drives BDNF/TrkB signal transduction (Liu et al., 2015), while α-syn can interrupt it (Kang et al., 2017). The detrimental effects of declining NE in PD may be compounded by the inhibition of BDNF-mediated neuroprotection by α-syn. This could contribute to the low serum BDNF levels that negatively correlate with motor impairment in later PD (Scalzo et al., 2010).

Neuroinflammation is a cardinal feature of PD and experimentally, both α-syn overexpression and lesion of the LC neurons result in inflammation (Theodore et al., 2008; Watson et al., 2012; Yao et al., 2015). While the experimental outcomes are currently inconsistent, it is clear that NE can modulate microglia activation status (Fujita et al., 1998; Gyoneva and Traynelis, 2013; Johnson et al., 2013). The decline in brain NE, increase in synucleinopathy, and subsequent modulation of microglia may contribute to the chronic inflammation found in PD brain tissue. Such inflammation is sufficient to induce parkinsonian neurodegeneration (Duffy et al., 2018; Song et al., 2018).

While the brain was once believed to be “immune privileged,” the entry of peripheral immune cells through the BBB is now a well-established feature of PD. Numerous immune cell populations are responsive to NE, and its deficiency in the periphery would diminish what seems to be a largely anti-inflammatory regulatory influence. This could promote exaggerated pro-inflammatory immune responses systemically. If the peripheral immune cells were recruited to the brain, reduced local NE levels combined with synuclein pathology would serve to augment and sustain inflammatory activity.

The physiological effects of neuroimmune interactions both centrally and peripherally are myriad, and their subtleties are just beginning to be appreciated and studied in detail. They may have the potential, however, to offer new therapeutic approaches for disorders such as PD for which effective treatments remain elusive. Future research evaluating the incidence of PD among individuals taking β-AR blockers (Mittal et al., 2017), for instance, and the rate of disease progression in PD patients treated with drugs that raise peripheral NE levels, such as droxidopa, could reveal new information about the role of NE in PD pathology.

## Author Contributions

LB contributed to conception and wrote the first draft of the manuscript. MH wrote sections of the manuscript. All authors contributed to manuscript revision and read and approved the submitted version.

## Conflict of Interest Statement

The authors declare that the research was conducted in the absence of any commercial or financial relationships that could be construed as a potential conflict of interest.

## References

[B1] Aarsland D. Andersen K. Larsen J. P. Lolk A. Kragh-Sorensen P. (2003). Prevalence and characteristics of dementia in Parkinson disease: an 8-year prospective study. *Arch. Neurol.* 60 387–392. 10.1001/archneur.60.3.387 12633150

[B2] Abbott R. D. Ross G. W. White L. R. Tanner C. M. Masaki K. H. Nelson J. S. (2005). Excessive daytime sleepiness and subsequent development of Parkinson disease. *Neurology* 65 1442–1446. 10.1212/01.wnl.0000183056.89590.0d 16275833

[B3] Agnati L. F. Zoli M. Stromberg I. Fuxe K. (1995). Intercellular communication in the brain: wiring versus volume transmission. *Neuroscience* 69 711–726. 10.1016/0306-4522(95)00308-6 8596642

[B4] Aston-Jones G. Rajkowski J. Cohen J. (1999). Role of locus coeruleus in attention and behavioral flexibility. *Biol. Psychiatry* 46 1309–1320. 10.1016/S0006-3223(99)00140-7 10560036

[B5] Aston-Jones G. Shipley M. T. Chouvet G. Ennis M. van Bockstaele E. Pieribone V. (1991). Afferent regulation of locus coeruleus neurons: anatomy, physiology and pharmacology. *Prog. Brain Res.* 88 47–75. 10.1016/S0079-6123(08)63799-1 1687622

[B6] Barrenschee M. Zorenkov D. Bottner M. Lange C. Cossais F. Scharf A. B. (2017). Distinct pattern of enteric phospho-alpha-synuclein aggregates and gene expression profiles in patients with Parkinson’s disease. *Acta Neuropathol. Commun.* 5:1. 10.1186/s40478-016-0408-2 28057070 PMC5217296

[B7] Bharani K. L. Derex R. Granholm A. C. Ledreux A. (2017). A noradrenergic lesion aggravates the effects of systemic inflammation on the hippocampus of aged rats. *PLoS One* 12:e0189821. 10.1371/journal.pone.0189821 29261743 PMC5736222

[B8] Bingham B. McFadden K. Zhang X. Bhatnagar S. Beck S. Valentino R. (2011). Early adolescence as a critical window during which social stress distinctly alters behavior and brain norepinephrine activity. *Neuropsychopharmacology* 36 896–909. 10.1038/npp.2010.229 21178981 PMC3055730

[B9] Blum-Degen D. Muller T. Kuhn W. Gerlach M. Przuntek H. Riederer P. (1995). Interleukin-1 beta and interleukin-6 are elevated in the cerebrospinal fluid of Alzheimer’s and de novo Parkinson’s disease patients. *Neurosci. Lett.* 202 17–20. 10.1016/0304-3940(95)12192-7 8787820

[B10] Boyko A. Melnikov M. Zhetishev R. Pashenkov M. (2016). The role of biogenic amines in the regulation of interaction between the immune and nervous systems in multiple Sclerosis. *Neuroimmunomodulation* 23 217–223. 10.1159/000449167 27710965

[B11] Braak E. Sandmann-Keil D. Rub U. Gai W. P. de Vos R. A. Steur E. N. (2001). alpha-synuclein immunopositive Parkinson’s disease-related inclusion bodies in lower brain stem nuclei. *Acta Neuropathol.* 101 195–201. 11307617 10.1007/s004010000247

[B12] Braak H. Del Tredici K. Rub U. de Vos R. A. Jansen Steur E. N. Braak E. (2003). Staging of brain pathology related to sporadic Parkinson’s disease. *Neurobiol. Aging* 24 197–211. 10.1016/S0197-4580(02)00065-9 12498954

[B13] Brochard V. Combadiere B. Prigent A. Laouar Y. Perrin A. Beray-Berthat V. (2009). Infiltration of CD4+ lymphocytes into the brain contributes to neurodegeneration in a mouse model of Parkinson disease. *J. Clin. Invest.* 119 182–192. 10.1172/JCI36470 19104149 PMC2613467

[B14] Brunnstrom H. Friberg N. Lindberg E. Englund E. (2011). Differential degeneration of the locus coeruleus in dementia subtypes. *Clin. Neuropathol.* 30 104–110. 10.5414/NPP30104 21545773

[B15] Brunskole Hummel I. Reinartz M. T. Kalble S. Burhenne H. Schwede F. Buschauer A. (2013). Dissociations in the effects of beta2-adrenergic receptor agonists on cAMP formation and superoxide production in human neutrophils: support for the concept of functional selectivity. *PLoS One* 8:e64556. 10.1371/journal.pone.0064556 23741338 PMC3669315

[B16] Buchman A. S. Nag S. Shulman J. M. Lim A. S. VanderHorst V. G. Leurgans S. E. (2012). Locus coeruleus neuron density and parkinsonism in older adults without Parkinson’s disease. *Mov. Disord.* 27 1625–1631. 10.1002/mds.25142 23038629 PMC3628555

[B17] Bucsek M. J. Giridharan T. MacDonald C. R. Hylander B. L. Repasky E. A. (2018). An overview of the role of sympathetic regulation of immune responses in infectious disease and autoimmunity. *Int. J. Hyperthermia* 34 135–143. 10.1080/02656736.2017.1411621 29498310 PMC6309867

[B18] Bucsek M. J. Qiao G. MacDonald C. R. Giridharan T. Evans L. Niedzwecki B. (2017). Beta-Adrenergic signaling in mice housed at standard temperatures suppresses an effector phenotype in CD8(+) T cells and undermines checkpoint inhibitor therapy. *Cancer Res.* 77 5639–5651. 10.1158/0008-5472.CAN-17-0546 28819022 PMC5645237

[B19] Burre J. Sharma M. Tsetsenis T. Buchman V. Etherton M. R. Sudhof T. C. (2010). Alpha-synuclein promotes SNARE-complex assembly in vivo and in vitro. *Science* 329 1663–1667. 10.1126/science.1195227 20798282 PMC3235365

[B20] Carvajal Gonczi C. M. Tabatabaei Shafiei M. East A. Martire E. Maurice-Ventouris M. H. I. Darlington P. J. (2017). Reciprocal modulation of helper Th1 and Th17 cells by the beta2-adrenergic receptor agonist drug terbutaline. *FEBS J.* 284 3018–3028. 10.1111/febs.14166 28710773

[B21] Casacchia M. Zamponi A. Squitieri G. Meco G. (1975). Treatment of anxiety in Parkinson’s disease with bromazepam. *Riv. Neurol.* 45 326–338. 7014

[B22] Castren E. Thoenen H. Lindholm D. (1995). Brain-derived neurotrophic factor messenger RNA is expressed in the septum, hypothalamus and in adrenergic brain stem nuclei of adult rat brain and is increased by osmotic stimulation in the paraventricular nucleus. *Neuroscience* 64 71–80. 10.1016/0306-4522(94)00386-J 7708216

[B23] Chalermpalanupap T. Schroeder J. P. Rorabaugh J. M. Liles L. C. Lah J. J. Levey A. I. (2018). Locus Coeruleus Ablation Exacerbates Cognitive Deficits, Neuropathology, and Lethality in P301S Tau Transgenic Mice. *J. Neurosci.* 38 74–92. 10.1523/JNEUROSCI.1483-17.2017 29133432 PMC5761438

[B24] Chan-Palay V. Asan E. (1989). Alterations in catecholamine neurons of the locus coeruleus in senile dementia of the Alzheimer type and in Parkinson’s disease with and without dementia and depression. *J. Comp. Neurol.* 287 373–392. 10.1002/cne.902870308 2570794

[B25] Chaudhuri K. R. Schapira A. H. (2009). Non-motor symptoms of Parkinson’s disease: dopaminergic pathophysiology and treatment. *Lancet Neurol.* 8 464–474. 10.1016/S1474-4422(09)70068-7 19375664

[B26] Chen G. Le Y. Zhou L. Gong L. Li X. Li Y. (2016). Dexmedetomidine inhibits maturation and function of human cord blood-derived dendritic cells by interfering with synthesis and secretion of IL-12 and IL-23. *PLoS One* 11:e0153288. 10.1371/journal.pone.0153288 27054340 PMC4824534

[B27] Chen M. J. Nguyen T. V. Pike C. J. Russo-Neustadt A. A. (2007). Norepinephrine induces BDNF and activates the PI-3K and MAPK cascades in embryonic hippocampal neurons. *Cell. Signal.* 19 114–128. 10.1016/j.cellsig.2006.05.028 16876982

[B28] Chen X. Huddleston D. E. Langley J. Ahn S. Barnum C. J. Factor S. A. (2014). Simultaneous imaging of locus coeruleus and substantia nigra with a quantitative neuromelanin MRI approach. *Magn. Reson. Imaging* 32 1301–1306. 10.1016/j.mri.2014.07.003 25086330

[B29] Cho M. (2014). Pacemaker’s burden. *Nat. Neurosci.* 17:755. 10.1038/nn0614-755 24866040

[B30] Chui H. C. Mortimer J. A. Slager U. Zarow C. Bondareff W. Webster D. D. (1986). Pathologic correlates of dementia in Parkinson’s disease. *Arch. Neurol.* 43 991–995. 10.1001/archneur.1986.00520100013007 3753274

[B31] Comella C. L. Nardine T. M. Diederich N. J. Stebbins G. T. (1998). Sleep-related violence, injury, and REM sleep behavior disorder in Parkinson’s disease. *Neurology* 51 526–529. 10.1212/WNL.51.2.526 9710029

[B32] Cosentino M. Fietta A. M. Ferrari M. Rasini E. Bombelli R. Carcano E. (2007). Human CD4+CD25+ regulatory T cells selectively express tyrosine hydroxylase and contain endogenous catecholamines subserving an autocrine/paracrine inhibitory functional loop. *Blood* 109 632–642. 10.1182/blood-2006-01-028423 16985181

[B33] Curtis A. L. Leiser S. C. Snyder K. Valentino R. J. (2012). Predator stress engages corticotropin-releasing factor and opioid systems to alter the operating mode of locus coeruleus norepinephrine neurons. *Neuropharmacology* 62 1737–1745. 10.1016/j.neuropharm.2011.11.020 22210331 PMC3269562

[B34] den Hartog J. W. Bethlem J. (1960). The distribution of Lewy bodies in the central and autonomic nervous systems in idiopathic paralysis agitans. *J. Neurol. Neurosurg. Psychiatry* 23 283–290. 10.1136/jnnp.23.4.283 13711997 PMC497426

[B35] Devauges V. Sara S. J. (1991). Memory retrieval enhancement by locus coeruleus stimulation: evidence for mediation by beta-receptors. *Behav. Brain Res.* 43 93–97. 10.1016/S0166-4328(05)80056-7 1650233

[B36] Devos D. Lebouvier T. Lardeux B. Biraud M. Rouaud T. Pouclet H. (2013). Colonic inflammation in Parkinson’s disease. *Neurobiol. Dis.* 50 42–48. 10.1016/j.nbd.2012.09.007 23017648

[B37] Dickson D. W. Fujishiro H. DelleDonne A. Menke J. Ahmed Z. Klos K. J. (2008). Evidence that incidental Lewy body disease is pre-symptomatic Parkinson’s disease. *Acta Neuropathol.* 115 437–444. 10.1007/s00401-008-0345-7 18264713

[B38] Duffy M. F. Collier T. J. Patterson J. R. Kemp C. J. Luk K. C. Tansey M. G. (2018). Lewy body-like alpha-synuclein inclusions trigger reactive microgliosis prior to nigral degeneration. *J. Neuroinflammation* 15:129. 10.1186/s12974-018-1171-z 29716614 PMC5930695

[B39] Eidson L. N. Kannarkat G. T. Barnum C. J. Chang J. Chung J. Caspell-Garcia C. (2017). Candidate inflammatory biomarkers display unique relationships with alpha-synuclein and correlate with measures of disease severity in subjects with Parkinson’s disease. *J. Neuroinflammation* 14:164. 10.1186/s12974-017-0935-1 28821274 PMC5563061

[B40] Eldrup E. Mogensen P. Jacobsen J. Pakkenberg H. Christensen N. J. (1995). CSF and plasma concentrations of free norepinephrine, dopamine, 3,4-dihydroxyphenylacetic acid (DOPAC), 3,4-dihydroxyphenylalanine (DOPA), and epinephrine in Parkinson’s disease. *Acta Neurol. Scand.* 92 116–121. 10.1111/j.1600-0404.1995.tb01023.x 7484057

[B41] Elizan T. S. Sroka H. Maker H. Smith H. Yahr M. D. (1986). Dementia in idiopathic Parkinson’s disease. *Variables associated with its occurrence in* 203 patients. *J. Neural Transm.* 65 285–302. 10.1007/BF01249089 3086503

[B42] Eskow Jaunarajs K. L. Angoa-Perez M. Kuhn D. M. Bishop C. (2011). Potential mechanisms underlying anxiety and depression in Parkinson’s disease: consequences of l-DOPA treatment. *Neurosci. Biobehav. Rev.* 35 556–564. 10.1016/j.neubiorev.2010.06.007 20615430 PMC2987522

[B43] Estrada L. D. Agac D. Farrar J. D. (2016). Sympathetic neural signaling via the beta2-adrenergic receptor suppresses T-cell receptor-mediated human and mouse CD8(+) T-cell effector function. *Eur. J. Immunol.* 46 1948–1958. 10.1002/eji.201646395 27222010 PMC5241047

[B44] Farrand A. Q. Helke K. L. Gregory R. A. Gooz M. Hinson V. K. Boger H. A. (2017). Vagus nerve stimulation improves locomotion and neuronal populations in a model of Parkinson’s disease. *Brain Stimul* 10 1045–1054. 10.1016/j.brs.2017.08.008 28918943 PMC5675746

[B45] Fearnley J. M. Lees A. J. (1991). Ageing and Parkinson’s disease: substantia nigra regional selectivity. *Brain* 114(Pt 5), 2283–2301. 10.1093/brain/114.5.2283 1933245

[B46] Freedman R. Foote S. L. Bloom F. E. (1975). Histochemical characterization of a neocortical projection of the nucleus locus coeruleus in the squirrel monkey. *J. Comp. Neurol.* 164 209–231. 10.1002/cne.901640205 810499

[B47] Fujita H. Tanaka J. Maeda N. Sakanaka M. (1998). Adrenergic agonists suppress the proliferation of microglia through beta 2-adrenergic receptor. *Neurosci. Lett.* 242 37–40. 10.1016/S0304-3940(98)00003-2 9509999

[B48] Gagnon J. F. Bedard M. A. Fantini M. L. Petit D. Panisset M. Rompre S. (2002). REM sleep behavior disorder and REM sleep without atonia in Parkinson’s disease. *Neurology* 59 585–589. 10.1212/WNL.59.4.585 12196654

[B49] Gao H. M. Zhang F. Zhou H. Kam W. Wilson B. Hong J. S. (2011). Neuroinflammation and alpha-synuclein dysfunction potentiate each other, driving chronic progression of neurodegeneration in a mouse model of Parkinson’s disease. *Environ. Health Perspect.* 119 807–814. 10.1289/ehp.1003013 21245015 PMC3114815

[B50] German D. C. Manaye K. F. White C. L. III Woodward D. J. McIntire D. D. Smith W. K. (1992). Disease-specific patterns of locus coeruleus cell loss. *Ann. Neurol.* 32 667–676. 10.1002/ana.410320510 1449247

[B51] Giasson B. I. Duda J. E. Quinn S. M. Zhang B. Trojanowski J. Q. Lee V. M. (2002). Neuronal alpha-synucleinopathy with severe movement disorder in mice expressing A53T human alpha-synuclein. *Neuron* 34 521–533. 10.1016/S0896-6273(02)00682-7 12062037

[B52] Gjerstad M. D. Wentzel-Larsen T. Aarsland D. Larsen J. P. (2007). Insomnia in Parkinson’s disease: frequency and progression over time. *J. Neurol. Neurosurg. Psychiatry* 78 476–479. 10.1136/jnnp.2006.100370 17098844 PMC2117851

[B53] Goldstein D. S. Eldadah B. A. Holmes C. Pechnik S. Moak J. Saleem A. (2005). Neurocirculatory abnormalities in Parkinson disease with orthostatic hypotension: independence from levodopa treatment. *Hypertension* 46 1333–1339. 10.1161/01.HYP.0000188052.69549.e4 16216982

[B54] Goldstein D. S. Holmes C. Sharabi Y. (2012). Cerebrospinal fluid biomarkers of central catecholamine deficiency in Parkinson’s disease and other synucleinopathies. *Brain* 135(Pt 6), 1900–1913. 10.1093/brain/aws055 22451506 PMC3359749

[B55] Goldstein D. S. Holmes C. Sharabi Y. Brentzel S. Eisenhofer G. (2003). Plasma levels of catechols and metanephrines in neurogenic orthostatic hypotension. *Neurology* 60 1327–1332. 10.1212/01.WNL.0000058766.46428.F3 12707437

[B56] Gonera E. G. van’t Hof M. Berger H. J. van Weel C. Horstink M. W. (1997). Symptoms and duration of the prodromal phase in Parkinson’s disease. *Mov. Disord.* 12 871–876. 10.1002/mds.870120607 9399209

[B57] Gosain A. Gamelli R. L. DiPietro L. A. (2009). Norepinephrine-mediated suppression of phagocytosis by wound neutrophils. *J. Surg. Res.* 152 311–318. 10.1016/j.jss.2008.05.001 18952237 PMC2683017

[B58] Grailer J. J. Haggadone M. D. Sarma J. V. Zetoune F. S. Ward P. A. (2014). Induction of M2 regulatory macrophages through the beta2-adrenergic receptor with protection during endotoxemia and acute lung injury. *J. Innate Immun.* 6 607–618. 10.1159/000358524 24642449 PMC4159611

[B59] Gray M. T. Woulfe J. M. (2015). Striatal blood-brain barrier permeability in Parkinson’s disease. *J. Cereb. Blood Flow Metab.* 35 747–750. 10.1038/jcbfm.2015.32 25757748 PMC4420870

[B60] Grzanna R. Molliver M. E. (1980). The locus coeruleus in the rat: an immunohistochemical delineation. *Neuroscience* 5 21–40. 10.1016/0306-4522(80)90068-8 6988734

[B61] Gyoneva S. Traynelis S. F. (2013). Norepinephrine modulates the motility of resting and activated microglia via different adrenergic receptors. *J. Biol. Chem.* 288 15291–15302. 10.1074/jbc.M113.458901 23548902 PMC3663549

[B62] Halliday G. M. Li Y. W. Blumbergs P. C. Joh T. H. Cotton R. G. Howe P. R. (1990). Neuropathology of immunohistochemically identified brainstem neurons in Parkinson’s disease. *Ann. Neurol.* 27 373–385. 10.1002/ana.410270405 1972319

[B63] Haniu M. Montestruque S. Bures E. J. Talvenheimo J. Toso R. Lewis-Sandy S. (1997). Interactions between brain-derived neurotrophic factor and the TRKB receptor. Identification of two ligand binding domains in soluble TRKB by affinity separation and chemical cross-linking. *J. Biol. Chem.* 272 25296–25303. 10.1074/jbc.272.40.25296 9312147

[B64] Harle P. Pongratz G. Albrecht J. Tarner I. H. Straub R. H. (2008). An early sympathetic nervous system influence exacerbates collagen-induced arthritis via CD4+CD25+ cells. *Arthritis Rheum.* 58 2347–2355. 10.1002/art.23628 18668589

[B65] Harms A. S. Thome A. D. Yan Z. Schonhoff A. M. Williams G. P. Li X. (2018). Peripheral monocyte entry is required for alpha-Synuclein induced inflammation and Neurodegeneration in a model of Parkinson disease. *Exp. Neurol.* 300 179–187. 10.1016/j.expneurol.2017.11.010 29155051 PMC5759972

[B66] Hayes G. M. Woodroofe M. N. Cuzner M. L. (1987). Microglia are the major cell type expressing MHC class II in human white matter. *J. Neurol. Sci.* 80 25–37. 10.1016/0022-510X(87)90218-8 3302117

[B67] Healy D. G. Abou-Sleiman P. M. Ozawa T. Lees A. J. Bhatia K. Ahmadi K. R. (2004). A functional polymorphism regulating dopamine beta-hydroxylase influences against Parkinson’s disease. *Ann. Neurol.* 55 443–446. 10.1002/ana.20063 14991826

[B68] Hely M. A. Reid W. G. Adena M. A. Halliday G. M. Morris J. G. (2008). The Sydney multicenter study of Parkinson’s disease: the inevitability of dementia at 20 years. *Mov. Disord.* 23 837–844. 10.1002/mds.21956 18307261

[B69] Heneka M. T. Galea E. Gavriluyk V. Dumitrescu-Ozimek L. Daeschner J. O’Banion M. K. (2002). Noradrenergic depletion potentiates beta -amyloid-induced cortical inflammation: implications for Alzheimer’s disease. *J. Neurosci.* 22 2434–2442. 10.1523/JNEUROSCI.22-07-02434.2002 11923407 PMC6758307

[B70] Henrich M. T. Geibl F. F. Lee B. Chiu W. H. Koprich J. B. Brotchie J. M. (2018). A53T-alpha-synuclein overexpression in murine locus coeruleus induces Parkinson’s disease-like pathology in neurons and glia. *Acta Neuropathol. Commun.* 6:39. 10.1186/s40478-018-0541-1 29747690 PMC5946574

[B71] Herve J. Dubreil L. Tardif V. Terme M. Pogu S. Anegon I. (2013). beta2-Adrenoreceptor agonist inhibits antigen cross-presentation by dendritic cells. *J. Immunol.* 190 3163–3171. 10.4049/jimmunol.1201391 23420884

[B72] Hirsch E. Graybiel A. M. Agid Y. A. (1988). Melanized dopaminergic neurons are differentially susceptible to degeneration in Parkinson’s disease. *Nature* 334 345–348. 10.1038/334345a0 2899295

[B73] Hobson J. A. McCarley R. W. Wyzinski P. W. (1975). Sleep cycle oscillation: reciprocal discharge by two brainstem neuronal groups. *Science* 189 55–58. 10.1126/science.1094539 1094539

[B74] Hoenen C. Gustin A. Birck C. Kirchmeyer M. Beaume N. Felten P. (2016). Alpha-synuclein proteins promote pro-inflammatory cascades in microglia: stronger effects of the A53T Mutant. *PLoS One* 11:e0162717. 10.1371/journal.pone.0162717 27622765 PMC5021287

[B75] Holmqvist S. Chutna O. Bousset L. Aldrin-Kirk P. Li W. Bjorklund T. (2014). Direct evidence of Parkinson pathology spread from the gastrointestinal tract to the brain in rats. *Acta Neuropathol.* 128 805–820. 10.1007/s00401-014-1343-6 25296989

[B76] Houser M. C. Chang J. Factor S. A. Molho E. S. Zabetian C. P. Hill-Burns E. M. (2018). stool immune profiles evince gastrointestinal inflammation in Parkinson’s disease. *Mov. Disord.* 33 793–804. 10.1002/mds.27326 29572994 PMC5992021

[B77] Houser M. C. Tansey M. G. (2017). The gut-brain axis: is intestinal inflammation a silent driver of Parkinson’s disease pathogenesis? *NPJ Parkinsons Dis.* 3:3. 10.1038/s41531-016-0002-0 28649603 PMC5445611

[B78] Howells D. W. Porritt M. J. Wong J. Y. Batchelor P. E. Kalnins R. Hughes A. J. (2000). Reduced BDNF mRNA expression in the Parkinson’s disease substantia nigra. *Exp. Neurol.* 166 127–135. 10.1006/exnr.2000.7483 11031089

[B79] Huang H. W. Fang X. X. Wang X. Q. Peng Y. P. Qiu Y. H. (2015). Regulation of differentiation and function of helper T cells by lymphocyte-derived catecholamines via alpha(1)- and beta(2)-adrenoceptors. *Neuroimmunomodulation* 22 138–151. 10.1159/000360579 24800755

[B80] Hunsley M. S. Palmiter R. D. (2003). Norepinephrine-deficient mice exhibit normal sleep-wake states but have shorter sleep latency after mild stress and low doses of amphetamine. *Sleep* 26 521–526. 12938804

[B81] Hurst J. H. LeWitt P. A. Burns R. S. Foster N. L. Lovenberg W. (1985). CSF dopamine-beta-hydroxylase activity in Parkinson’s disease. *Neurology* 35 565–568. 10.1212/WNL.35.4.565 3982644

[B82] Hyman C. Hofer M. Barde Y. A. Juhasz M. Yancopoulos G. D. Squinto S. P. (1991). BDNF is a neurotrophic factor for dopaminergic neurons of the substantia nigra. *Nature* 350 230–232. 10.1038/350230a0 2005978

[B83] Inoue T. Tanaka S. Okusa M. D. (2017). Neuroimmune Interactions in Inflammation and Acute Kidney Injury. *Front. Immunol.* 8:945. 10.3389/fimmu.2017.00945 28848551 PMC5552660

[B84] Iranzo A. Molinuevo J. L. Santamaria J. Serradell M. Marti M. J. Valldeoriola F. (2006). Rapid-eye-movement sleep behaviour disorder as an early marker for a neurodegenerative disorder: a descriptive study. *Lancet Neurol.* 5 572–577. 10.1016/S1474-4422(06)70476-8 16781987

[B85] Isaias I. U. Trujillo P. Summers P. Marotta G. Mainardi L. Pezzoli G. (2016). Neuromelanin Imaging and Dopaminergic Loss in Parkinson’s Disease. *Front. Aging Neurosci.* 8:196 10.3389/fnagi.2016.00196 PMC4992725 27597825

[B86] Iversen L. L. Rossor M. N. Reynolds G. P. Hills R. Roth M. Mountjoy C. Q. (1983). Loss of pigmented dopamine-beta-hydroxylase positive cells from locus coeruleus in senile dementia of Alzheimer’s type. *Neurosci. Lett.* 39 95–100. 10.1016/0304-3940(83)90171-4 6633940

[B87] Iwai A. Masliah E. Yoshimoto M. Ge N. Flanagan L. de Silva H. A. (1995). The precursor protein of non-A beta component of Alzheimer’s disease amyloid is a presynaptic protein of the central nervous system. *Neuron* 14 467–475. 10.1016/0896-6273(95)90302-X 7857654

[B88] Javoy-Agid F. Ruberg M. Taquet H. Bokobza B. Agid Y. Gaspar P. (1984). Biochemical neuropathology of Parkinson’s disease. *Adv. Neurol.* 40 189–198. 6695594

[B89] Jenner P. (2003). Oxidative stress in Parkinson’s disease. *Ann. Neurol* 53(Suppl. 3), S26–S36. 10.1002/ana.10483 12666096

[B90] Johnson J. D. Zimomra Z. R. Stewart L. T. (2013). Beta-adrenergic receptor activation primes microglia cytokine production. *J. Neuroimmunol.* 254 161–164. 10.1016/j.jneuroim.2012.08.007 22944319

[B91] Joling M. van den Heuvel O. A. Berendse H. W. Booij J. Vriend C. (2018). Serotonin transporter binding and anxiety symptoms in Parkinson’s disease. *J. Neurol. Neurosurg. Psychiatry* 89 89–94. 10.1136/jnnp-2017-316193 28899958

[B92] Jones B. E. Yang T. Z. (1985). The efferent projections from the reticular formation and the locus coeruleus studied by anterograde and retrograde axonal transport in the rat. *J. Comp. Neurol.* 242 56–92. 10.1002/cne.902420105 2416786

[B93] Kalaitzakis M. E. Gentleman S. M. Pearce R. K. (2013). Disturbed sleep in Parkinson’s disease: anatomical and pathological correlates. *Neuropathol. Appl. Neurobiol.* 39 644–653. 10.1111/nan.12024 23363035

[B94] Kalinin S. Feinstein D. L. Xu H. L. Huesa G. Pelligrino D. A. Galea E. (2006). Degeneration of noradrenergic fibres from the locus coeruleus causes tight-junction disorganisation in the rat brain. *Eur. J. Neurosci.* 24 3393–3400. 10.1111/j.1460-9568.2006.05223.x 17229089

[B95] Kang S. S. Zhang Z. Liu X. Manfredsson F. P. Benskey M. J. Cao X. (2017). TrkB neurotrophic activities are blocked by alpha-synuclein, triggering dopaminergic cell death in Parkinson’s disease. *Proc. Natl. Acad. Sci. U.S.A.* 114 10773–10778. 10.1073/pnas.1713969114 28923922 PMC5635931

[B96] Kannarkat G. T. Boss J. M. Tansey M. G. (2013). The role of innate and adaptive immunity in Parkinson’s disease. *J Parkinsons Dis* 3 493–514. 10.3233/JPD-130250 24275605 PMC4102262

[B97] Kelly L. P. Carvey P. M. Keshavarzian A. Shannon K. M. Shaikh M. Bakay R. A. (2014). Progression of intestinal permeability changes and alpha-synuclein expression in a mouse model of Parkinson’s disease. *Mov. Disord.* 29 999–1009. 10.1002/mds.25736 24898698 PMC4050039

[B98] Keren N. I. Taheri S. Vazey E. M. Morgan P. S. Granholm A. C. Aston-Jones G. S. (2015). Histologic validation of locus coeruleus MRI contrast in post-mortem tissue. *Neuroimage* 113 235–245. 10.1016/j.neuroimage.2015.03.020 25791783 PMC4649944

[B99] Khan M. M. Sansoni P. Engleman E. G. Melmon K. L. (1985). Pharmacologic effects of autacoids on subsets of T cells. Regulation of expression/function of histamine-2 receptors by a subset of suppressor cells. *J Clin Invest* 75 1578–1583. 10.1172/JCI111863 2860125 PMC425498

[B100] Kilbourn M. R. Sherman P. Abbott L. C. (1998). Reduced MPTP neurotoxicity in striatum of the mutant mouse tottering. *Synapse* 30 205–210. 10.1002/(SICI)1098-2396(199810)30:2<205::AID-SYN10>3.0.CO;2-0 9723790

[B101] Kim S. Park J. M. Moon J. Choi H. J. (2014). Alpha-synuclein interferes with cAMP/PKA-dependent upregulation of dopamine beta-hydroxylase and is associated with abnormal adaptive responses to immobilization stress. *Exp. Neurol.* 252 63–74. 10.1016/j.expneurol.2013.11.009 24252179

[B102] Kim S. S. Moon K. R. Choi H. J. (2011). Interference of alpha-synuclein with cAMP/PKA-dependent CREB signaling for tyrosine hydroxylase gene expression in SK-N-BE(2)C cells. *Arch. Pharm. Res.* 34 837–845. 10.1007/s12272-011-0518-0 21656370

[B103] Kim Y. S. Joh T. H. (2006). Microglia, major player in the brain inflammation: their roles in the pathogenesis of Parkinson’s disease. *Exp. Mol. Med.* 38 333–347. 10.1038/emm.2006.40 16953112

[B104] Kin N. W. Sanders V. M. (2006). It takes nerve to tell T and B cells what to do. *J. Leukoc. Biol.* 79 1093–1104. 10.1189/jlb.1105625 16531560

[B105] Klein C. Schlossmacher M. G. (2006). The genetics of Parkinson disease: implications for neurological care. *Nat. Clin. Pract. Neurol.* 2 136–146. 10.1038/ncpneuro0126 16932540

[B106] Klimek V. Stockmeier C. Overholser J. Meltzer H. Y. Kalka S. Dilley G. (1997). Reduced levels of norepinephrine transporters in the locus coeruleus in major depression. *J. Neurosci.* 17 8451–8458. 10.1523/JNEUROSCI.17-21-08451.1997 9334417 PMC6573768

[B107] Koppel I. Jaanson K. Klasche A. Tuvikene J. Tiirik T. Parn A. (2018). Dopamine cross-reacts with adrenoreceptors in cortical astrocytes to induce BDNF expression, CREB signaling and morphological transformation. *Glia* 66 206–216. 10.1002/glia.23238 28983964

[B108] Kortekaas R. Leenders K. L. van Oostrom J. C. Vaalburg W. Bart J. Willemsen A. T. (2005). Blood-brain barrier dysfunction in parkinsonian midbrain in vivo. *Ann. Neurol.* 57 176–179. 10.1002/ana.20369 15668963

[B109] Laumet G. Edralin J. D. Chiang A. C. Dantzer R. Heijnen C. J. Kavelaars A. (2018). Resolution of inflammation-induced depression requires T lymphocytes and endogenous brain interleukin-10 signaling. *Neuropsychopharmacology* 10.1038/s41386-018-0154-1 [Epub ahead of print]. 30054585 PMC6224384

[B110] Lin C. H. Lin J. W. Liu Y. C. Chang C. H. Wu R. M. (2015). Risk of Parkinson’s disease following anxiety disorders: a nationwide population-based cohort study. *Eur. J. Neurol.* 22 1280–1287. 10.1111/ene.12740 26031920

[B111] Liu X. Ye K. Weinshenker D. (2015). Norepinephrine Protects against Amyloid-beta Toxicity via TrkB. *J. Alzheimers Dis.* 44 251–260. 10.3233/JAD-141062 25208620 PMC4714587

[B112] Liu Y. Rui X. X. Shi H. Qiu Y. H. Peng Y. P. (2018). Norepinephrine Inhibits Th17 Cells via beta2-Adrenergic Receptor (beta2-AR) Signaling in a Mouse Model of Rheumatoid Arthritis. *Med. Sci. Monit.* 24 1196–1204. 10.12659/MSM.906184 29485127 PMC5839072

[B113] Loavenbruck A. Sandroni P. (2015). Neurogenic orthostatic hypotension: roles of norepinephrine deficiency in its causes, its treatment, and future research directions. *Curr. Med. Res. Opin.* 31 2095–2104. 10.1185/03007995.2015.1087988 26373628

[B114] Lorton D. Bellinger D. L. (2015). Molecular mechanisms underlying beta-adrenergic receptor-mediated cross-talk between sympathetic neurons and immune cells. *Int. J. Mol. Sci.* 16 5635–5665. 10.3390/ijms16035635 25768345 PMC4394497

[B115] Mann D. M. Yates P. O. (1983). Pathological basis for neurotransmitter changes in Parkinson’s disease. *Neuropathol. Appl. Neurobiol.* 9 3–19. 10.1111/j.1365-2990.1983.tb00320.x 6133229

[B116] Masliah E. Rockenstein E. Veinbergs I. Mallory M. Hashimoto M. Takeda A. (2000). Dopaminergic loss and inclusion body formation in alpha-synuclein mice: implications for neurodegenerative disorders. *Science* 287 1265–1269. 10.1126/science.287.5456.1265 10678833

[B117] Mavridis M. Degryse A. D. Lategan A. J. Marien M. R. Colpaert F. C. (1991). Effects of locus coeruleus lesions on parkinsonian signs, striatal dopamine and substantia nigra cell loss after 1-methyl-4-phenyl-1,2,3,6-tetrahydropyridine in monkeys: a possible role for the locus coeruleus in the progression of Parkinson’s disease. *Neuroscience* 41 507–523. 10.1016/0306-4522(91)90345-O 1870701

[B118] McAlees J. W. Smith L. T. Erbe R. S. Jarjoura D. Ponzio N. M. Sanders V. M. (2011). Epigenetic regulation of beta2-adrenergic receptor expression in T(H)1 and T(H)2 cells. *Brain Behav. Immun.* 25 408–415. 10.1016/j.bbi.2010.10.019 21047549 PMC3073579

[B119] McCall J. G. Al-Hasani R. Siuda E. R. Hong D. Y. Norris A. J. Ford C. P. (2015). CRH Engagement of the Locus Coeruleus Noradrenergic System Mediates Stress-Induced Anxiety. *Neuron* 87 605–620. 10.1016/j.neuron.2015.07.002 26212712 PMC4529361

[B120] McGuire S. O. Ling Z. D. Lipton J. W. Sortwell C. E. Collier T. J. Carvey P. M. (2001). Tumor necrosis factor alpha is toxic to embryonic mesencephalic dopamine neurons. *Exp. Neurol.* 169 219–230. 10.1006/exnr.2001.7688 11358437

[B121] Mello-Carpes P. B. da Silva de Vargas L. Gayer M. C. Roehrs R. Izquierdo I. (2016). Hippocampal noradrenergic activation is necessary for object recognition memory consolidation and can promote BDNF increase and memory persistence. *Neurobiol. Learn. Mem.* 127 84–92. 10.1016/j.nlm.2015.11.014 26691781

[B122] Merola A. Romagnolo A. Rosso M. Lopez-Castellanos J. R. Wissel B. D. Larkin S. (2016). Orthostatic hypotension in Parkinson’s disease: does it matter if asymptomatic? *Parkinsonism Relat. Disord.* 33 65–71. 10.1016/j.parkreldis.2016.09.013 27641792

[B123] Miller R. M. Kiser G. L. Kaysser-Kranich T. Casaceli C. Colla E. Lee M. K. (2007). Wild-type and mutant alpha-synuclein induce a multi-component gene expression profile consistent with shared pathophysiology in different transgenic mouse models of PD. *Exp. Neurol.* 204 421–432. 10.1016/j.expneurol.2006.12.005 17254569

[B124] Mittal S. Bjornevik K. Im D. S. Flierl A. Dong X. Locascio J. J. (2017). beta2-Adrenoreceptor is a regulator of the alpha-synuclein gene driving risk of Parkinson’s disease. *Science* 357 891–898. 10.1126/science.aaf3934 28860381 PMC5761666

[B125] Mogi M. Harada M. Riederer P. Narabayashi H. Fujita K. Nagatsu T. (1994). Tumor necrosis factor-alpha (TNF-alpha) increases both in the brain and in the cerebrospinal fluid from parkinsonian patients. *Neurosci. Lett.* 165 208–210. 10.1016/0304-3940(94)90746-3 8015728

[B126] Moriguchi S. Yamada M. Takano H. Nagashima T. Takahata K. Yokokawa K. (2017). Norepinephrine Transporter in Major Depressive Disorder: a PET Study. *Am. J. Psychiatry* 174 36–41. 10.1176/appi.ajp.2016.15101334 27631962

[B127] Mount M. P. Lira A. Grimes D. Smith P. D. Faucher S. Slack R. (2007). Involvement of interferon-gamma in microglial-mediated loss of dopaminergic neurons. *J. Neurosci.* 27 3328–3337. 10.1523/JNEUROSCI.5321-06.2007 17376993 PMC6672486

[B128] Mouton P. R. Pakkenberg B. Gundersen H. J. Price D. L. (1994). Absolute number and size of pigmented locus coeruleus neurons in young and aged individuals. *J. Chem. Neuroanat.* 7 185–190. 10.1016/0891-0618(94)90028-0 7848573

[B129] Murphy A. C. Lalor S. J. Lynch M. A. Mills K. H. (2010). Infiltration of Th1 and Th17 cells and activation of microglia in the CNS during the course of experimental autoimmune encephalomyelitis. *Brain Behav. Immun.* 24 641–651. 10.1016/j.bbi.2010.01.014 20138983

[B130] Nag S. Harik S. I. (1987). Cerebrovascular permeability to horseradish peroxidase in hypertensive rats: effects of unilateral locus ceruleus lesion. *Acta Neuropathol.* 73 247–253. 10.1007/BF00686618 3113166

[B131] Niimi Y. Ieda T. Hirayama M. Koike Y. Sobue G. Hasegawa Y. (1999). Clinical and physiological characteristics of autonomic failure with Parkinson’s disease. *Clin. Auton. Res* 9 139–144. 10.1007/BF02281627 10454060

[B132] Nimmerjahn A. Kirchhoff F. Helmchen F. (2005). Resting microglial cells are highly dynamic surveillants of brain parenchyma in vivo. *Science* 308 1314–1318. 10.1126/science.1110647 15831717

[B133] Noguchi T. Nakagome K. Kobayashi T. Ueda Y. Soma T. Nakamoto H. (2015). Effect of beta2-adrenergic agonists on eosinophil adhesion, superoxide anion generation, and degranulation. *Allergol. Int.* 64(Suppl.), S46–S53. 10.1016/j.alit.2015.05.009 26344080

[B134] Noh H. Yu M. R. Kim H. J. Lee J. H. Park B. W. Wu I. H. (2017). Beta 2-adrenergic receptor agonists are novel regulators of macrophage activation in diabetic renal and cardiovascular complications. *Kidney Int.* 92 101–113. 10.1016/j.kint.2017.02.013 28396116 PMC5483383

[B135] Nuti A. Ceravolo R. Piccinni A. Dell’Agnello G. Bellini G. Gambaccini G. (2004). Psychiatric comorbidity in a population of Parkinson’s disease patients. *Eur. J. Neurol.* 11 315–320. 10.1111/j.1468-1331.2004.00781.x 15142224

[B136] Ohno M. Yoshimatsu A. Kobayashi M. Watanabe S. (1997). Noradrenergic DSP-4 lesions aggravate impairment of working memory produced by hippocampal muscarinic blockade in rats. *Pharmacol. Biochem. Behav.* 57 257–261. 10.1016/S0091-3057(96)00353-X 9164580

[B137] Pamphlett R. (2014). Uptake of environmental toxicants by the locus ceruleus: a potential trigger for neurodegenerative, demyelinating and psychiatric disorders. *Med. Hypotheses* 82 97–104. 10.1016/j.mehy.2013.11.016 24315447

[B138] Parkinson J. (2002). An essay on the shaking palsy. 1817. *J. Neuropsychiatry Clin. Neurosci.* 14 223–236; discussion 222. 10.1176/jnp.14.2.223 11983801

[B139] Pavese N. Rivero-Bosch M. Lewis S. J. Whone A. L. Brooks D. J. (2011). Progression of monoaminergic dysfunction in Parkinson’s disease: a longitudinal 18F-dopa PET study. *Neuroimage* 56 1463–1468. 10.1016/j.neuroimage.2011.03.012 21396455

[B140] Pifl C. Kish S. J. Hornykiewicz O. (2012). Thalamic noradrenaline in Parkinson’s disease: deficits suggest role in motor and non-motor symptoms. *Mov. Disord.* 27 1618–1624. 10.1002/mds.25109 23038412 PMC4533102

[B141] Pisani V. Stefani A. Pierantozzi M. Natoli S. Stanzione P. Franciotta D. (2012). Increased blood-cerebrospinal fluid transfer of albumin in advanced Parkinson’s disease. *J Neuroinflammation* 9:188. 10.1186/1742-2094-9-188 22870899 PMC3441323

[B142] Pocock J. M. Kettenmann H. (2007). Neurotransmitter receptors on microglia. *Trends Neurosci.* 30 527–535. 10.1016/j.tins.2007.07.007 17904651

[B143] Pongratz G. Anthofer J. M. Melzer M. Anders S. Grassel S. Straub R. H. (2014). IL-7 receptor alpha expressing B cells act proinflammatory in collagen-induced arthritis and are inhibited by sympathetic neurotransmitters. *Ann. Rheum. Dis.* 73 306–312. 10.1136/annrheumdis-2012-202944 23505234

[B144] Pongratz G. Melzer M. Straub R. H. (2012). The sympathetic nervous system stimulates anti-inflammatory B cells in collagen-type II-induced arthritis. *Ann. Rheum. Dis.* 71 432–439. 10.1136/ard.2011.153056 21953335

[B145] Postuma R. B. Gagnon J. F. Vendette M. Fantini M. L. Massicotte-Marquez J. Montplaisir J. (2009). Quantifying the risk of neurodegenerative disease in idiopathic REM sleep behavior disorder. *Neurology* 72 1296–1300. 10.1212/01.wnl.0000340980.19702.6e 19109537 PMC2828948

[B146] Punsoni M. Friedman J. H. Resnick M. Donahue J. E. Yang D. F. Stopa E. G. (2017). Enteric Pathologic Manifestations of Alpha-Synucleinopathies. *Appl. Immunohistochem. Mol. Morphol.* 10.1097/PAI.0000000000000613 [Epub ahead of print]. 29189256

[B147] Qiao G. Chen M. Bucsek M. J. Repasky E. A. Hylander B. L. (2018). Adrenergic Signaling: a targetable checkpoint limiting development of the antitumor immune response. *Front. Immunol.* 9:164. 10.3389/fimmu.2018.00164 29479349 PMC5812031

[B148] Qin X. Y. Zhang S. P. Cao C. Loh Y. P. Cheng Y. (2016). Aberrations in peripheral inflammatory cytokine levels in Parkinson disease: a systematic review and meta-analysis. *JAMA Neurol.* 73 1316–1324. 10.1001/jamaneurol.2016.2742 27668667

[B149] Ramer-Quinn D. S. Baker R. A. Sanders V. M. (1997). Activated T helper 1 and T helper 2 cells differentially express the beta-2-adrenergic receptor: a mechanism for selective modulation of T helper 1 cell cytokine production. *J. Immunol.* 159 4857–4867. 9366411

[B150] Rana A. Q. Qureshi A. R. M. Shamli Oghli Y. Saqib Y. Mohammed B. Sarfraz Z. (2018). Decreased sleep quality in Parkinson’s patients is associated with higher anxiety and depression prevalence and severity, and correlates with pain intensity and quality. *Neurol. Res* 10.1080/01616412.2018.1462880 [Epub ahead of print]. 29663852

[B151] Ravina B. Camicioli R. Como P. G. Marsh L. Jankovic J. Weintraub D. (2007). The impact of depressive symptoms in early Parkinson disease. *Neurology* 69 342–347. 10.1212/01.wnl.0000268695.63392.10 17581943 PMC2031220

[B152] Reijnders J. S. Ehrt U. Weber W. E. Aarsland D. Leentjens A. F. (2008). A systematic review of prevalence studies of depression in Parkinson’s disease. *Mov. Disord.* 23 183–189;quiz313. 10.1002/mds.21803 17987654

[B153] Remy P. Doder M. Lees A. Turjanski N. Brooks D. (2005). Depression in Parkinson’s disease: loss of dopamine and noradrenaline innervation in the limbic system. *Brain* 128(Pt 6), 1314–1322. 10.1093/brain/awh445 15716302

[B154] Ressler K. J. Nemeroff C. B. (2001). Role of norepinephrine in the pathophysiology of neuropsychiatric disorders. *CNS Spectr.* 6 663–666. 10.1017/S1092852900001358 15520614

[B155] Riether C. Kavelaars A. Wirth T. Pacheco-Lopez G. Doenlen R. Willemen H. (2011). Stimulation of beta(2)-adrenergic receptors inhibits calcineurin activity in CD4(+) T cells via PKA-AKAP interaction. *Brain Behav. Immun.* 25 59–66. 10.1016/j.bbi.2010.07.248 20674738

[B156] Rite I. Machado A. Cano J. Venero J. L. (2007). Blood-brain barrier disruption induces in vivo degeneration of nigral dopaminergic neurons. *J. Neurochem.* 101 1567–1582. 10.1111/j.1471-4159.2007.04567.x 17437543

[B157] Rommelfanger K. S. Edwards G. L. Freeman K. G. Liles L. C. Miller G. W. Weinshenker D. (2007). Norepinephrine loss produces more profound motor deficits than MPTP treatment in mice. *Proc. Natl. Acad. Sci. U.S.A.* 104 13804–13809. 10.1073/pnas.0702753104 17702867 PMC1959463

[B158] Rommelfanger K. S. Weinshenker D. Miller G. W. (2004). Reduced MPTP toxicity in noradrenaline transporter knockout mice. *J. Neurochem.* 91 1116–1124. 10.1111/j.1471-4159.2004.02785.x 15569255

[B159] Ross G. W. Petrovitch H. Abbott R. D. Tanner C. M. Popper J. Masaki K. (2008). Association of olfactory dysfunction with risk for future Parkinson’s disease. *Ann. Neurol.* 63 167–173. 10.1002/ana.21291 18067173

[B160] Roth T. L. Nayak D. Atanasijevic T. Koretsky A. P. Latour L. L. McGavern D. B. (2014). Transcranial amelioration of inflammation and cell death after brain injury. *Nature* 505 223–228. 10.1038/nature12808 24317693 PMC3930079

[B161] Rye D. B. Bliwise D. L. Dihenia B. Gurecki P. (2000). FAST TRACK: daytime sleepiness in Parkinson’s disease. *J. Sleep Res.* 9 63–69. 10.1046/j.1365-2869.2000.00201.x 10733691

[B162] Sanchez-Guajardo V. Barnum C. J. Tansey M. G. Romero-Ramos M. (2013). Neuroimmunological processes in Parkinson’s disease and their relation to alpha-synuclein: microglia as the referee between neuronal processes and peripheral immunity. *ASN Neuro* 5 113–139. 10.1042/AN20120066 23506036 PMC3639751

[B163] Scalzo P. Kummer A. Bretas T. L. Cardoso F. Teixeira A. L. (2010). Serum levels of brain-derived neurotrophic factor correlate with motor impairment in Parkinson’s disease. *J. Neurol.* 257 540–545. 10.1007/s00415-009-5357-2 19847468

[B164] Schenck C. H. Boeve B. F. Mahowald M. W. (2013). Delayed emergence of a parkinsonian disorder or dementia in 81% of older men initially diagnosed with idiopathic rapid eye movement sleep behavior disorder: a 16-year update on a previously reported series. *Sleep Med.* 14 744–748. 10.1016/j.sleep.2012.10.009 23347909

[B165] Seiffert K. Hosoi J. Torii H. Ozawa H. Ding W. Campton K. (2002). Catecholamines inhibit the antigen-presenting capability of epidermal Langerhans cells. *J. Immunol.* 168 6128–6135. 10.4049/jimmunol.168.12.6128 12055224

[B166] Senard J. M. Valet P. Durrieu G. Berlan M. Tran M. A. Montastruc J. L. (1990). Adrenergic supersensitivity in parkinsonians with orthostatic hypotension. *Eur. J. Clin. Invest.* 20 613–619. 10.1111/j.1365-2362.1990.tb01909.x 1964123

[B167] Shibayama-Imazu T. Okahashi I. Omata K. Nakajo S. Ochiai H. Nakai Y. (1993). Cell and tissue distribution and developmental change of neuron specific 14 kDa protein (phosphoneuroprotein 14). *Brain Res.* 622 17–25. 10.1016/0006-8993(93)90796-P 7694766

[B168] Shulman L. M. Taback R. L. Rabinstein A. A. Weiner W. J. (2002). Non-recognition of depression and other non-motor symptoms in Parkinson’s disease. *Parkinsonism. Relat. Disord.* 8 193–197. 10.1016/S1353-8020(01)00015-3 12039431

[B169] Sixel-Doring F. Trautmann E. Mollenhauer B. Trenkwalder C. (2011). Associated factors for REM sleep behavior disorder in Parkinson disease. *Neurology* 77 1048–1054. 10.1212/WNL.0b013e31822e560e 21832215

[B170] Smith M. C. Ellgring H. Oertel W. H. (1997). Sleep disturbances in Parkinson’s disease patients and spouses. *J. Am. Geriatr. Soc.* 45 194–199. 10.1111/j.1532-5415.1997.tb04506.x 9033518

[B171] Song S. Jiang L. Oyarzabal E. A. Wilson B. Li Z. Shih Y. I. (2018). Loss of brain norepinephrine elicits neuroinflammation-mediated oxidative injury and selective caudo-rostral neurodegeneration. *Mol. Neurobiol.* 10.1007/s12035-018-1235-1 [Epub ahead of print]. 30051353 PMC6348128

[B172] Spillantini M. G. Schmidt M. L. Lee V. M. Trojanowski J. Q. Jakes R. Goedert M. (1997). Alpha-synuclein in Lewy bodies. *Nature* 388 839–840. 10.1038/42166 9278044

[B173] Spina M. B. Squinto S. P. Miller J. Lindsay R. M. Hyman C. (1992). Brain-derived neurotrophic factor protects dopamine neurons against 6-hydroxydopamine and N-methyl-4-phenylpyridinium ion toxicity: involvement of the glutathione system. *J. Neurochem.* 59 99–106. 10.1111/j.1471-4159.1992.tb08880.x 1613515

[B174] Srinivasan J. Schmidt W. J. (2003). Potentiation of parkinsonian symptoms by depletion of locus coeruleus noradrenaline in 6-hydroxydopamine-induced partial degeneration of substantia nigra in rats. *Eur. J. Neurosci.* 17 2586–2592. 10.1046/j.1460-9568.2003.02684.x 12823465

[B175] Stefanis L. (2012). alpha-Synuclein in Parkinson’s disease. *Cold Spring Harb Perspect Med* 2 a009399. 10.1101/cshperspect.a009399 22355802 PMC3281589

[B176] Stein M. B. Heuser I. J. Juncos J. L. Uhde T. W. (1990). Anxiety disorders in patients with Parkinson’s disease. *Am. J. Psychiatry* 147 217–220. 10.1176/ajp.147.2.217 2301664

[B177] Stokholm M. G. Danielsen E. H. Hamilton-Dutoit S. J. Borghammer P. (2016). Pathological alpha-synuclein in gastrointestinal tissues from prodromal Parkinson disease patients. *Ann. Neurol.* 79 940–949. 10.1002/ana.24648 27015771

[B178] Stolzenberg E. Berry D. Yang Lee E. Y. Kroemer A. Kaufman S (2017). A Role for Neuronal Alpha-Synuclein in Gastrointestinal Immunity. *J. Innate Immun.* 9 456–463. 10.1159/000477990 28651250 PMC5865636

[B179] Strosberg A. D. (1993). Structure, function, and regulation of adrenergic receptors. *Protein Sci.* 2 1198–1209. 10.1002/pro.5560020802 8401205 PMC2142449

[B180] Sulzer D. Alcalay R. N. Garretti F. Cote L. Kanter E. Agin-Liebes J. (2017). T cells from patients with Parkinson’s disease recognize alpha-synuclein peptides. *Nature* 546 656–661. 10.1038/nature22815 28636593 PMC5626019

[B181] Szot P. White S. S. Greenup J. L. Leverenz J. B. Peskind E. R. Raskind M. A. (2006). Compensatory changes in the noradrenergic nervous system in the locus ceruleus and hippocampus of postmortem subjects with Alzheimer’s disease and dementia with Lewy bodies. *J. Neurosci.* 26 467–478. 10.1523/JNEUROSCI.4265-05.2006 16407544 PMC6674412

[B182] Takamoto T. Hori Y. Koga Y. Toshima H. Hara A. Yokoyama M. M. (1991). Norepinephrine inhibits human natural killer cell activity in vitro. *Int. J. Neurosci.* 58 127–131. 10.3109/00207459108987189 1657810

[B183] Tanaka K. F. Kashima H. Suzuki H. Ono K. Sawada M. (2002). Existence of functional beta1- and beta2-adrenergic receptors on microglia. *J. Neurosci. Res.* 70 232–237. 10.1002/jnr.10399 12271472

[B184] Tansey M. G. Goldberg M. S. (2010). Neuroinflammation in Parkinson’s disease: its role in neuronal death and implications for therapeutic intervention. *Neurobiol. Dis.* 37 510–518. 10.1016/j.nbd.2009.11.004 19913097 PMC2823829

[B185] Theodore S. Cao S. McLean P. J. Standaert D. G. (2008). Targeted overexpression of human alpha-synuclein triggers microglial activation and an adaptive immune response in a mouse model of Parkinson disease. *J. Neuropathol. Exp. Neurol.* 67 1149–1158. 10.1097/NEN.0b013e31818e5e99 19018246 PMC2753200

[B186] Thobois S. Prange S. Sgambato-Faure V. Tremblay L. Broussolle E. (2017). Imaging the etiology of apathy, anxiety, and depression in Parkinson’s Disease: implication for treatment. *Curr. Neurol. Neurosci. Rep.* 17:76. 10.1007/s11910-017-0788-0 28822071

[B187] Tong J. Hornykiewicz O. Kish S. J. (2006). Inverse relationship between brain noradrenaline level and dopamine loss in Parkinson disease: a possible neuroprotective role for noradrenaline. *Arch. Neurol.* 63 1724–1728. 10.1001/archneur.63.12.1724 17172611

[B188] Toshimitsu M. Kamei Y. Ichinose M. Seyama T. Imada S. Iriyama T. (2018). Atomoxetine, a selective norepinephrine reuptake inhibitor, improves short-term histological outcomes after hypoxic-ischemic brain injury in the neonatal male rat. *Int. J. Dev. Neurosci.* 10.1016/j.ijdevneu.2018.03.011 [Epub ahead of print]. 29608930

[B189] Troadec J. D. Marien M. Darios F. Hartmann A. Ruberg M. Colpaert F. (2001). Noradrenaline provides long-term protection to dopaminergic neurons by reducing oxidative stress. *J. Neurochem.* 79 200–210. 10.1046/j.1471-4159.2001.00556.x 11595772

[B190] Troadec J. D. Marien M. Mourlevat S. Debeir T. Ruberg M. Colpaert F. (2002). Activation of the mitogen-activated protein kinase (ERK(1/2)) signaling pathway by cyclic AMP potentiates the neuroprotective effect of the neurotransmitter noradrenaline on dopaminergic neurons. *Mol. Pharmacol.* 62 1043–1052. 10.1124/mol.62.5.1043 12391266

[B191] Vida G. Pena G. Kanashiro A. Thompson-Bonilla Mdel R. Palange D. Deitch E. A. (2011). beta2-Adrenoreceptors of regulatory lymphocytes are essential for vagal neuromodulation of the innate immune system. *FASEB J.* 25 4476–4485. 10.1096/fj.11-191007 21840939 PMC3236627

[B192] Wang X. M. Zhang Y. G. Li A. L. Long Z. H. Wang D. Li X. X. (2016). Relationship between levels of inflammatory cytokines in the peripheral blood and the severity of depression and anxiety in patients with Parkinson’s disease. *Eur. Rev. Med. Pharmacol. Sci.* 20 3853–3856. 27735031

[B193] Watson M. B. Richter F. Lee S. K. Gabby L. Wu J. Masliah E. (2012). Regionally-specific microglial activation in young mice over-expressing human wildtype alpha-synuclein. *Exp. Neurol.* 237 318–334. 10.1016/j.expneurol.2012.06.025 22750327 PMC3443323

[B194] Wersinger C. Jeannotte A. Sidhu A. (2006). Attenuation of the norepinephrine transporter activity and trafficking via interactions with alpha-synuclein. *Eur. J. Neurosci.* 24 3141–3152. 10.1111/j.1460-9568.2006.05181.x 17156375

[B195] Whalen M. M. Bankhurst A. D. (1990). Effects of beta-adrenergic receptor activation, cholera toxin and forskolin on human natural killer cell function. *Biochem. J.* 272 327–331. 10.1042/bj2720327 2176460 PMC1149703

[B196] Wirth T. Westendorf A. M. Bloemker D. Wildmann J. Engler H. Mollerus S. (2014). The sympathetic nervous system modulates CD4(+)Foxp3(+) regulatory T cells via noradrenaline-dependent apoptosis in a murine model of lymphoproliferative disease. *Brain Behav. Immun.* 38 100–110. 10.1016/j.bbi.2014.01.007 24440144

[B197] Wu D. C. Jackson-Lewis V. Vila M. Tieu K. Teismann P. Vadseth C. (2002). Blockade of microglial activation is neuroprotective in the 1-methyl-4-phenyl-1,2,3,6-tetrahydropyridine mouse model of Parkinson disease. *J. Neurosci.* 22 1763–1771. 10.1523/JNEUROSCI.22-05-01763.2002 11880505 PMC6758858

[B198] Wu H. Chen J. Song S. Yuan P. Liu L. Zhang Y. (2016). beta2-adrenoceptor signaling reduction in dendritic cells is involved in the inflammatory response in adjuvant-induced arthritic rats. *Sci. Rep.* 6:24548. 10.1038/srep24548 27079168 PMC4832233

[B199] Xu L. Ding W. Stohl L. L. Zhou X. K. Azizi S. Chuang E. (2018). Regulation of T helper cell responses during antigen presentation by norepinephrine-exposed endothelial cells. *Immunology* 154 104–121. 10.1111/imm.12871 29164596 PMC5904699

[B200] Yao N. Wu Y. Zhou Y. Ju L. Liu Y. Ju R. (2015). Lesion of the locus coeruleus aggravates dopaminergic neuron degeneration by modulating microglial function in mouse models of Parkinsons disease. *Brain Res.* 1625 255–274. 10.1016/j.brainres.2015.08.032 26342895

[B201] Zarow C. Lyness S. A. Mortimer J. A. Chui H. C. (2003). Neuronal loss is greater in the locus coeruleus than nucleus basalis and substantia nigra in Alzheimer and Parkinson diseases. *Arch. Neurol.* 60 337–341. 10.1001/archneur.60.3.337 12633144

[B202] Zhang W. Wang T. Pei Z. Miller D. S. Wu X. Block M. L. (2005). Aggregated alpha-synuclein activates microglia: a process leading to disease progression in Parkinson’s disease. *FASEB J.* 19 533–542. 10.1096/fj.04-2751com 15791003

